# Supplementary material for: The causal role of circulating inflammatory markers in osteoporosis: a bidirectional Mendelian randomized study
Source: Front Immunol. 2024 Jul 18;15:1412298. doi: 10.3389/fimmu.2024.1412298 (PMC11291241; doi:10.3389/fimmu.2024.1412298)
Supplement: Supplementary file 1 [file DataSheet_1.zip › supplementary material/CIM-OSTEOPOROSIS /GCST90274811.csv_forest.pdf]

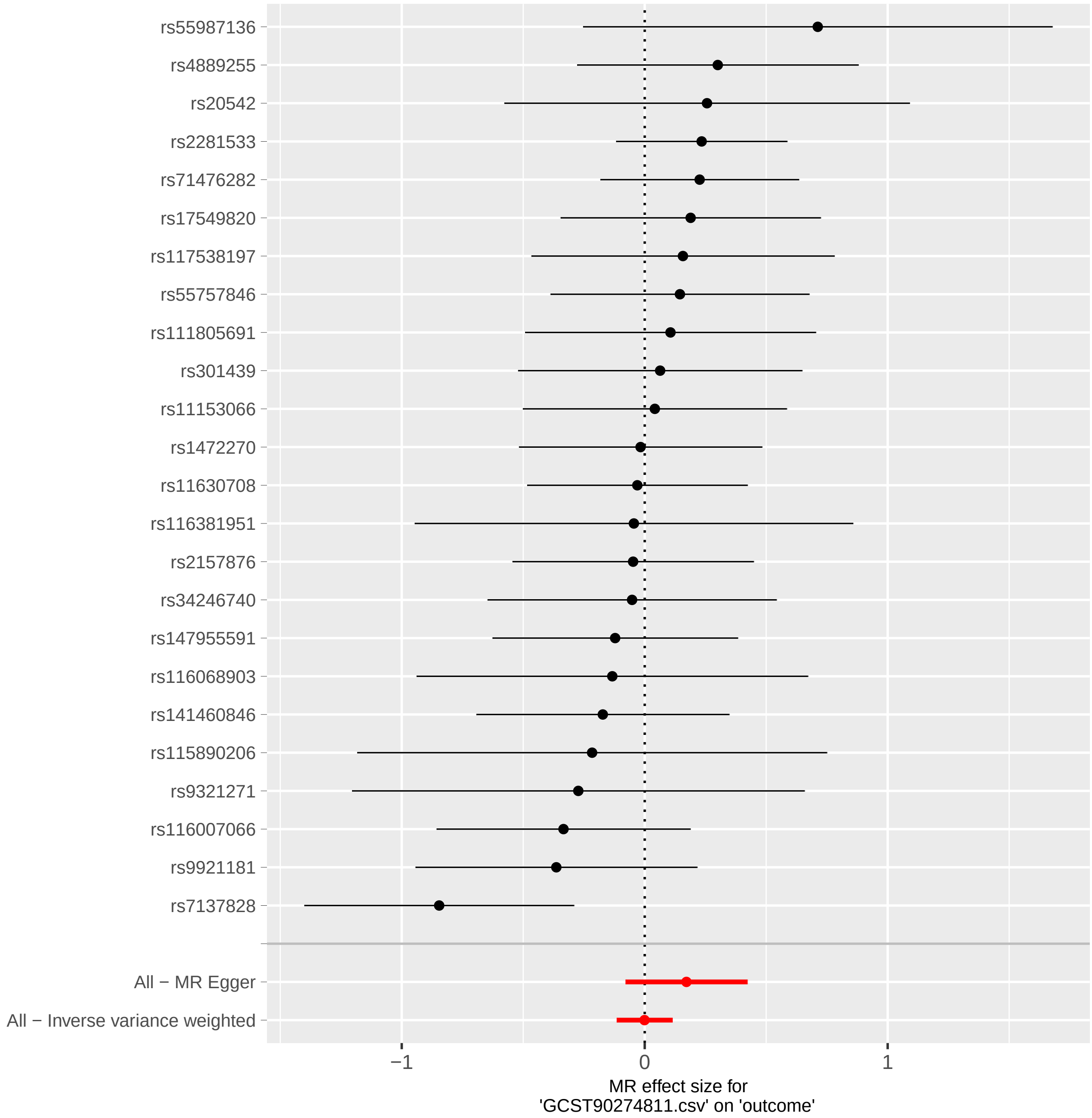

rs55987136
rs4889255
rs20542
rs2281533
rs71476282
rs17549820
rs117538197
rs55757846
rs111805691
rs301439
rs11153066
rs1472270
rs11630708
rs116381951
rs2157876
rs34246740
rs147955591
rs116068903
rs141460846
rs115890206
rs9321271
rs116007066
rs9921181
rs7137828
All − MR Egger
All − Inverse variance weighted
−1
0
1
MR effect size for
'GCST90274811.csv' on 'outcome'
